# Toward Accurate Estimation of Extractable Organic Fluorine by Analysis of Fluoride Contamination in Extracts Using Gas Chromatography

**DOI:** 10.1021/acs.analchem.5c07147

**Published:** 2026-04-02

**Authors:** Johannes Kikuchi-McIntosh, Malin Montelius, Gustav Sporre, David Bastviken, Teresia Svensson

**Affiliations:** † Department of Thematic Studies − Environmental Change, 4566 Linköping University, Linköping SE-581 83, Sweden; ‡ 52901 Swedish Geotechnical Institute (SGI), Linköping SE-581 93, Sweden

## Abstract

Extractable organic fluorine (EOF) analysis is used to assess fluorinated organic content in, e.g., water and solid sample extracts. EOF content is often determined by combustion ion chromatography (CIC). However, contamination from inorganic fluoride (F^–^) in extracts risks overestimating actual EOF. Despite this known interference, a standardized and optimized methodology to mitigate such contamination remains lacking. In this study, we evaluated the presence of F^–^ in EOF extracts and designed and validated a method for F^–^ determination in small aliquots (∼250 μL) of complex aqueous and solvent matrices. The method is based on derivatization of F^–^ to triphenylfluorosilane (TPSiF) and subsequent analysis with gas-chromatographic tandem mass spectrometry analysis (GC–MS/MS). Calibration was linear from 1 to 1000 μg L^–1^ (*R* ^2^ > 0.99), and the limit of detection and the limit of quantification were 0.98 and 4.5 μg F^–^ L^–1^, respectively. The relative standard deviation was below 7% across the calibrated range. A certified reference material (CRM River water) and F^–^-spiked minced meat were used to assess F^–^ recovery to 103 ± 3% (*p* = 0.10) and 95 ± 6% (*p* = 0.31) of certified/spiked concentrations. Analysis of sample extracts showed that (1) solid-phase extraction extracts of river water, (2) solvent extracts from aqueous-film-forming foam (AFFF)-impacted soil, and (3) extracts of a fish sample contained 29–49, 61 ± 4.7, and 17–90 μg F^–^ L^–1^, respectively. F^–^ accounted for 3–59% of the measured EOF, indicating a substantial overestimation. Therefore, the analysis and discount of F^–^ in EOF extracts are essential for accurate EOF determination by CIC.

## Graphical abstract

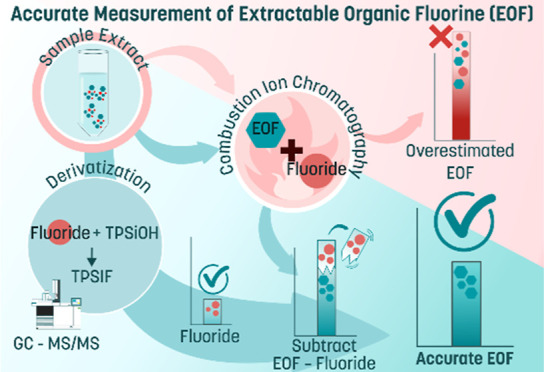

## Introduction

Per- and polyfluoroalkyl substances (PFAS) represent one of the most worrying global health- and environmental threats of today.^1^ Given their widespread use^2^ and environmental persistence,^3^ PFAS has been suggested to constitute a new planetary boundary^4,5^ that humanity has exceeded.^6^ Knowledge of human PFAS exposure first emerged during the 1960s when diverging fluorine (F) concentrations were observed in human blood plasma, and it was revealed that some of the analytical methods used only measured F^–^, while others measured total fluorine (TF), i.e., both inorganic and organic F.^7–11^ Combination of these methodological differences allowed for estimation of the organic fluorine (OF) fraction as the difference between TF and F^–^ ^7,8,12,13^ (TF minus F^–^). Eventually, parts of the OF fraction in blood plasma were isolated, and its major constituent, the PFAS perfluorooctanesulfonic acid (PFOS), was determined by F NMR.^14^ While F NMR confirmed the structure of PFOS in human blood, it was the ability to distinguish F^–^ from OF in the blood F mass balance that led to this discovery.

Given the plethora of PFAS in use today (∼14 000 compounds^15^), it is common to analyze aliquots of PFAS extracts for extractable organic fluorine (EOF) using combustion ion chromatography (CIC)^16–21^ to estimate potentially unidentified PFAS. However, organic extracts for PFAS/EOF analysis can contain inorganic fluorinated anions such as tetrafluoroborate (BF_4_^–^) and hexafluorophosphate (PF_6_^–^) that, if not accounted for by target analysis, will lead to overestimation of the OF fraction.^22^ F^–^ in extracts could also constitute a source of overestimation of the OF fraction and despite being an integral part of estimating OF in the past,^7–10^ most contemporary studies neglect analysis of F^–^ in extracts for EOF measurements.^16–21^

F removal efficiencies of 95–99.995% in extraction procedures have been reported during method development for PFAS/EOF extraction.^16,20,27,33^ However, as F^–^ can be much more abundant than OF, extraction efficiencies at, e.g., 95–>99% can still result in substantial F^–^ contamination compared to OF concentrations. Given the inherent heterogeneity of environmental samples, it is uncertain whether F^–^ removal efficiency can be assumed to be independent and unaffected by different matrices and matrix effects. If not, residual F^–^ might remain in sample extracts and contribute to the apparent EOF. Such uncertainties can be mitigated by quantifying F^–^ concentrations in aliquots of extracts and subtract from analyzed F-concentrations from, e.g., CIC to yield a more accurate EOF.

Measurements of F^–^ are typically made with IC or F^–^ selective electrodes (FSE).^23^ For IC, sample solutions are commonly injected via a peristaltic pump on the IC autosampler, and FSE’s need to be immersed in the sample solution. Consequently, these methods require large volumes of sample (several milliliters) for analysis. This is problematic considering that extract volumes for PFAS analysis rarely exceed 1–2 mL.^24–27^ Furthermore, IC measurements suffer from interferences from coeluting compounds such as formate, acetate, and lactate.^28^ The use of FSE’s lead to other issues as they are cross-sensitive to hydroxide ions (OH^–^) which necessitates the use of total ionic strength adjustment buffers (TISAB) to minimize interference from OH^–^ and to adjust pH to shift the chemical equilibrium HF ⇌ F^–^ toward fluoride.^23,29^ The use of TISAB buffers will further dilute the extract and fluoride, potentially below the detection limits. Standard addition methods have been used to decrease the detection limits of FSEs^30,31^ but such methods are in general tedious and time-consuming since they require repeated measurements on the same sample for accurate fluoride determinations. These issues make IC and FSE determinations problematic because many PFAS extraction procedures involve manipulating pH with acids and bases (hydrochloric acid (HCl)^27^ or formic acid (HCOOH)^32^ and sodium- or ammonium-hydroxide (NaOH^32,33^ or NH_4_OH^27,28,34^ ).

Finding other F^–^ analysis methods that are rapid and fit-for-purpose could help mitigate potential overestimation of the EOF. Gas chromatography (GC) with various detectors has been used for F^–^ determination and could circumvent some of the issues mentioned above. F^–^ needs to be derivatized to a volatile form to be amenable for GC analysis.^35^ For example, conversion of F^–^ to silyl derivatives (R_*x*_–Si–F) under highly acidic conditions has provided high sensitivity and selectivity even with small sample volumes (<1 mL).^36–38^

While GC analysis potentially addresses issues with F^–^ measurements of EOF extracts, some aspects of the GC approach of F^–^ analysis require improvements for broader application and utility. For example, common use of concentrated perchloric acid (HClO_4_) during silylation poses a significant work hazard due to its oxidative power and risk of explosions,^39,40^ and finding alternatives would be preferable. A study using triphenylsilanol (TPSiOH) to derivatize F^–^ found that concentrated nitric acid (HNO_3_) produced similar derivatization efficiencies as HClO_4_.^38^ However, HClO_4_ was preferred due to lower F^–^ blank levels. This suggests that HNO_3_, a much weaker oxidizing agent, could be a viable alternative to HClO_4_ if background levels of F^–^ could be controlled.

Derivatization time is another important aspect for efficient workflows and method utility. For silyl derivatizations, reaction times vary from 5 to 150 min depending on the derivatization agent.^36–38,41,42^ Silylation reactions with very short reaction times require cooled conditions due to high volatility,^41^ but there are more suitable options. Derivatization of TPSiOH to triphenylfluorosilane (TPSiF) can be performed at room temperature in 1 h,^36,38^ making it both fast and easy to use. Previous related studies also suggest that the derivatization time can be optimized further.^38^

To conclude, simple F^–^ measurements in extracts could remove some uncertainties regarding EOF as analyzed with CIC, if appropriate methods were developed. Apart from fulfilling regular method validation criteria, such methods would have to be compatible with small sample volumes, complex environmental matrices, rapid in their execution, and preferably avoid reagents that pose unnecessary work safety hazards. As such, we set out to develop and optimize a rapid, easy-to-use derivatization procedure for conversion of F^–^ to TPSiF with HNO_3_ and subsequent analysis by GC–MS/MS. The aim was to use sample volumes smaller than 1 mL and derivatization times under 1 h. After validation, we explored F^–^ content in surface water-, aqueous-film-forming foam (AFFF)-impacted soil-, and fish extracts from various PFAS extraction procedures along with CIC measurements to determine EOF.

## Definitions

For clarity, we repeat here the definitions of the key acronyms we use for the various fluorine fractions: F^–^ refers to fluoride, that is, the inorganic, monatomic anion of fluorine that is measured by the developed GC–MS/MS method in this study. EF refers to extractable fluorine as measured by CIC (inorganic + OF). Finally, the EOF refers to EOF defined as EF minus F^–^.

## Experimental Section

### Chemicals

All chemicals used for method development and validation were of analytical grade or higher. Triphenylfluorosilane (TPSiF) was purchased from BLDpharm (97.95% purity), and the derivatizing agent triphenylsilanol (TPSiOH) was from Thermo Scientific (98% purity). All optimization tests were made on dilutions of the mixed anion standard SS-9195S 1000 μg mL^–1^ (Spectrascan, Teknolab) containing equal concentrations of F^–^, Cl^–^, Br^–^, I^–^, NO_3_^–^, NO_2_^–^, SO_4_^2–^, and PO_4_^3–^. The certified reference material (CRM) LGC6025 River Water—Anions (LGC Standards) was used during method validation (F^–^, Cl^–^, NO_3_^–^, and SO_4_^2–^; 1.248, 31.3, 38, and 66.2 mg L^–1^). Laboratory reagent-grade *n*-heptane (Fisher Chemical, >99% purity) was purchased from Fisher Scientific. See the Supporting Information for the other materials and chemicals used during the various extraction procedures.

### F^–^ Removal from Concentrated HNO_3_

Initial testing of the derivatization procedure revealed that concentrated HNO_3_ was a large source of F^–^ contamination. Pure water blank concentrations exceeded 1000 μg F^–^ L^–1^ using 3 mL concentrated of HNO_3_ (65%, EMSURE Supelco), 2 mL of ultrapure water, and 250 μL 0.015 M TPSiOH (data not shown) for derivatization. Similar results were found with the higher purity concentrated HNO_3_, EMSURE 65% Suprapur (with a stated F^–^ content of <1 ppm) (data not shown). Given that the acids with different purity gave similar results, it was decided to use the cheaper, lower grade purity acid but to try to develop procedures to remove F^–^ from it. Two approaches were tested: (a) derivatization of F^–^ in the acid to TPSiF and extraction with sequential additions of heptane prior to use and (b) mixing silica gel (SiO_2_, Molecular Sieve 3 Å (Thermo Scientific) or Silica Gel 60 μm (Supelco)) with the acid at acid-to-silica ratios 30:1 and ∼4:1, to facilitate the formation of surface –Si–F complexes, thereby removing F^–^ from the solution,^43^ see the Supporting Information for the theoretical basis of this approach. Testing revealed that mixing at an acid-to-silica ratio of ∼4:1 gave the best results but still needed improvement. Thus, a further iteration was performed where 40 mL of acid from the ∼4:1 treatment was mixed with an additional spatula of silica gel followed by vortexing, centrifugation, and derivatization. The initial testing scheme is summarized in Table 1, and the details on the testing protocol can be found in the Supporting Information.

**Table 1:** Initial Testing Scheme for Removal of F^–^ from Concentrated HNO_3_ by Derivatization to TPSiF with Subsequent Extraction, Surface Complexation with Molecular Sieve, or Surface Complexation with Silica Gel by Mixing Sieve or Gel with Concentrated HNO_3_ ^b^

Removal procedure	HNO_3_ (mL)	0.015 M TPSiOH (mL)/SiO_2_ (g)	Acid-to-silica ratio
F^–^ to TPSiF w. extraction^a^	9	1	-
molecular sieve 3 Å^a^	9	0.3	30:1
silica gel 60^a^	9	0.3	30:1
silica gel 60^a^	∼400	0.3	∼4:1

a All tests were carried out in triplicate.

b Performed as a single replicate with ∼400 mL of concentrated HNO_3_ and 121 g silica gel but analyzed in triplicate.

### Factor Optimization Scheme for Derivatization

After reducing the F^–^ content of HNO_3_, a full factorial design was used to optimize derivatization parameters: acid-to-sample ratio (mL mL^–1^), amount of added TPSiOH (μmol), and derivatization time (min). Each parameter was investigated at a low and high level; see Table 2 for investigated levels of each parameter. A center point was added in between the low and high levels of each parameter to investigate if there was a derivatization optimum between these levels. Levels were chosen according to previous literature on derivatization procedures.^36^ In total, this design gave 30 samples including 2^3^ = 8 test combinations of high/low levels in triplicates and center points with 6 replicates. The samples were randomized to two blocks, 15 samples for each block, because the maximum number of sample vials that could be processed at the same time for derivatization was 18. Randomization allowed us to account for possible variation arising from the different derivatization batches. This design was evaluated with a linear mixed effects model with block as random variable and ANOVA to find optimum parameter levels for derivatization efficiency. The center point was excluded from the linear model but was used to compare derivatization efficiency to the low and high levels. Once the factorial design was evaluated, it became clear that the highest level could have been set even higher, and as such additional testing with acid-to-sample ratio 3.3 and 4 were tested while keeping the amount of TPSiOH constant at 1 μmol. These additional tests were carried out at 20, 30, and 40 min derivatization time.

**Table 2:** Factorial Design for Optimization of Derivatization Efficiency of F^–^ to TPSiF^c^

	Acid-to-sample ratio (mL mL^–1^)^a^	TPSiOH (μmol)^a^ ^,^ ^b^	Derivatization time (min)^a^	HNO3/sample/TPSiOH (μL)
Factorial design	1	0.5	20	600:600:33
	1	0.5	60	600:600:33
	1	1	20	600:600:67
	1	1	60	600:600:67
	2	0.5	20	800:400:33
	2	0.5	60	800:400:33
	2	1	20	800:400:67
	2	1	60	800:400:67
Center point^b^	1.5	0.75	40	750:500:50
				
Additional tests	3	1	20	1000:300:67
	3	1	40	1000:300:67
	4	1	20	1000:250:67
	4	1	30	1000:250:67
	4	1	40	1000:250:67

a Combination of low and high variable levels performed in triplicate randomized to two blocks.

b Concentration of 0.015 mol L^–1^ TPSiOH.

c Performed in six replicates.

All data treatment and statistical analysis were performed in R. The package lme4 was used for the linear mixed effects model, and lmerTest and pbkrtest were used for type 3 ANOVA with the “Kenward-Roger” method for approximating the number of degrees of freedom.

### Method Validation

The method validation assessed linearity, accuracy, precision, limit of detection (LOD), and limit of quantification (LOQ). Precision tests were performed by derivatizing and analyzing five replicate samples at 5, 100, 500, and 1000 μg F^–^ L^–1^, respectively. Accuracy was assessed by analyzing F^–^ spiked minced meat samples extracted with water and the CRM River Water sample. LOD and LOQ were determined by analyzing 10 ultrapure water blanks and calculating the concentration corresponding to the mean blank signal plus three standard deviations of the mean (LOD) and the mean blank signal plus ten standard deviations of the mean (LOQ).

### Samples and Sample Preparation

To test the applicability of the method for F^–^ determination, we performed extraction procedures for PFAS/EOF on three types of samples: surface water (Svartå River), AFFF-contaminated soil, and the CRM IRMM 427 Pike-perch PFAS reference sample (sample ID 173) (see Table 3). The river water was extracted by tandem-solid-phase extraction (solid-phase extraction (SPE)) by stacking (from top to bottom) an Oasis WAX,^44^ ISOLUTE ENV+,^45^ and ISOLUTE 101 ^46^ column on top of each other. The soil was extracted with an alkaline/acidic MeOH procedure^27^ and the CRM Pike-perch sample with the QuEChERS*ER* ^47^ method (Quick, Easy, Cheap, Effective, Rugged, Safe^48^ extraction based on MeCN extraction, -*ER* implies that the method is more than QuEChERS) and alkaline/acidic MeOH.^27^ For details on the extraction procedures, see the Supporting Information section Extraction Procedures. We then investigated if F^–^ was present in the different extracts with the developed derivatization method and determined EF with CIC to establish F mass balances of the extracts (see the next section). The river water was sampled with a Ruttner sampler (2 L) at the mouth of the Svartå River as it enters lake Roxen north of Linköping, Sweden. The sampling depth was 1 m beneath the water surface. The soil sample came from an AFFF-impacted area (∑_17_PFAS = 195 ± 4 μg of PFAS kg^–1^, analysis by SGS Analytics Sweden AB) in proximity to an active fire station. The soil was classified as a silty sand based on its particle density distribution (SS-EN ISO 17892-4-2016) and was sampled from the surface to 40 cm depth and was thoroughly sieved <2 mm, homogenized, and split using a riffle splitter prior to sample preparation and extraction. The CRM Pike-perch sample was obtained from the European Commission Joint Research Centre Institute for Reference Materials and Measurements (EC JRC) and was thoroughly homogenized prior to use as per instructions for the reference material. Sample type, mode of extraction, and reference methods are compiled in Table 3.

**Table 3:** Samples and Extraction Procedures for the Determination of EOF and F^–^.

Sample	Extraction	Reference
Svartå River Water	SPE Oasis WAX	Miyake et al. (2007)^28^
	SPE ISOLUTE ENV+	Biotage (2020)^45,46^
	SPE ISOLUTE 101	Biotage (2020)^45,46^
AFFF soil	Alkaline/acidic MeOH	Nickerson et al. (2020)^a^ ^,^ ^27^
CRM Pike-perch	Alkaline/acidic MeOH	Nickerson et al. (2020)^a^ ^,^ ^27^
CRM Pike-perch	QuEChERSER-MeCN/H_2_O	Taylor and Sapozhnikova (2022)^a^ ^,^ ^47^

a With slight modification in this study.

Sample extract aliquots of 250 μL were evaporated to dryness and reconstituted in 250 μL of ultrapure water prior to derivatization with the optimized derivatization method for F^–^ determination with GC–MS/MS. For EF measurements, 20–100 μL of sample extract was added to quartz boats and subsequently combusted using hydropyrolysis and analyzed on the IC.

### Uncertainty

For each extraction procedure, three replicate sample extracts and three replicate extraction blanks were made. Measured F^–^ and EF in extracts were then corrected by subtracting the average concentrations of the extraction blanks. Blank-corrected F^–^ values were then subtracted from the obtained blank-corrected EF for each replicate. Averages, standard deviations, standard error of the mean (SEM), and relative standard error of the mean (RSE) of F^–^, EF, and EOF concentrations were then calculated for each extraction triplicate. Because subtractions of F^–^ from EF were made within each replicate, the final average EOF fractions (with standard deviations, SEMs, and RSEs) inherently account for the integrated errors arising from the analysis of both F^–^ and EF and the subsequent subtraction. Reported SEMs and RSEs (Table S6) therefore represent an empirical uncertainty measure of the mean F fractions of each extract.

### Instrumentation

The GC–MS/MS system was composed of an Agilent 8890 GC connected to Agilent 7010B triple quadrupole with an Agilent 7693 Autosampler. The GC column was an HP-5 ms Ultra Inert (30 m × 250 μm × 0.25 μm, length, inner diameter, film thickness). The He carrier-gas flow was set to 1 mL min^–1^, and a 0.2 μL split injection (split rate 20:1) was used with an inlet temperature of 325 °C. The GC-oven program started with a 0.5 min hold time at 40 °C, followed by a 60 °C min^–1^ increase to 280 °C with 1.5 min hold time. Finally, the temperature was increased to 300 °C at a rate of 60 °C min^–1^ with a 1.7 min hold time yielding a total run time of 8.7 min with ∼4 min re-equilibration between injections.

The transfer line temperature between the column and the mass spectrometer was set to 300 °C and the ion source temperature to 280 °C. Electron impact ionization at 70 eV was used for ionization, and parent and fragment ions were monitored using multireaction monitoring with N_2_ as collision gas. For the derivatized product, TPSiF, the following transitions were monitored *m*/*z* 278 → 201, 278 → 181 with 201 as the quantifier. The Agilent MassHunter Optimizer program was used to find the optimum collision energies for fragmentation.

CIC was performed using a Metrohm 930 Compact IC Flex instrument connected to the Metrohm 920 Absorber Module and Analytik Jena Combustion Module with the MMS 5000 Automatic Boat Drive autosampler. Instrument settings were combustion temperature 1050 °C, O_2_ gas flow of 300 mL min^–1^, Ar gas flow of 100 mL min^–1^, 0.2 mL min^–1^ water addition during combustion, the postcombustion time was set to 5 min, yielding a total combustion time of 9.6 min, the initial absorber solution volume was 3 mL ultrapure water, and the total absorber solution volume after combustion was ∼7.6 mL. For sample injection, ∼2.2 mL of the absorber solution was preconcentrated onto the preconcentration column (Metrosep A PCC 2 VHC 4.0) and eluted with 0.7 mL min^–1^ gradient elution flow with Eluent A (0.25 mM NaHCO_3_) and B (12 mM Na_2_CO_3_/10 mM NaOH/7.5% ethanol (v/v)) followed by analytical separation on the Metrosep A Supp 5 150/4.0. The IC calibrated range was 5–5000 μg F L^–1^, and the quality of the combustion step was controlled by combustion of the CRM River Water sample, a 0.5 ppm PFOS solution and IC standards.

## Results and Discussion

### F^–^ Contamination and Removal

Derivatization of F^–^ in the concentrated HNO_3_ followed by heptane extraction (TPSiF extraction) had the lowest capacity to reduce F^–^ levels in the acid, see Figure 1. The silica gel 30:1 treatment reduced F^–^ levels in the acid to 52 ± 25 μg F^–^ L^–1^ (mean ± SD), which was 4 times lower than that of the molecular sieve 30:1 treatment (206 ± 48 μg F^–^ L^–1^, Figure 1). Increasing the amount of silica to a ratio of 4:1 (Silica Gel 4:1) lowered the F^–^ background levels even further to 15 ± 9 μg of F^–^ L^–1^. The iteration with 40 mL of the 4:1 acid, mixed with an additional spatula of silica gel, gave the best result, ultimately lowering the F^–^ background levels to 0.7 ± 0.5 μg F^–^ L^–1^ (*n* = 10), which was as low as F^–^ levels in derivatized ultrapure water used for solution preparation and as the blank for the F^–^ analyses. Accordingly, given this lowest residual F^–^ in the iteration with 40 mL of 4:1 acid with additional silica gel additions (reaching levels below the F^–^ analysis blanks), this procedure was chosen for continued work in the derivatization optimization factorial design. A previous study also employing derivatization with TPSiOH reported F^–^ contamination, likely originating from the acid. In their case, it was perchloric acid with a blank sample contribution of ∼2.3 μg F^–^ L^–1^.^38^

**Figure 1:**
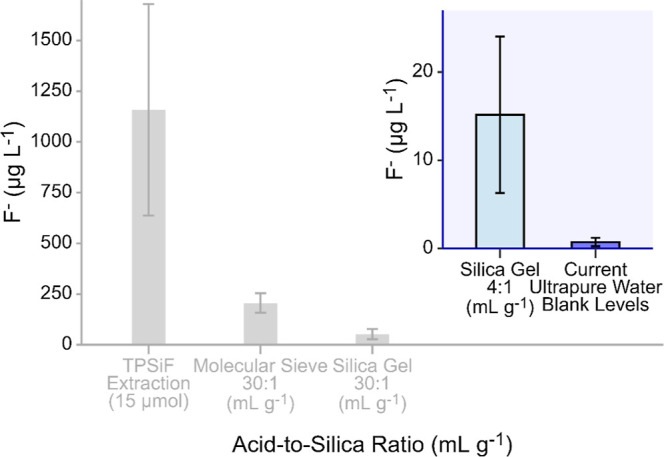
Remaining F^–^ in concentrated HNO_3_ after derivatization and extraction of TPSiF (TPSiF Extraction 15 μmol), mixing with molecular sieve at a ratio of 30:1 (Molecular Sieve 30:1), mixing with silica gel at a ratio of 30:1 and 4:1 (Silica Gel 30:1, Silica Gel 4:1). The current method blank levels (ultrapure water samples) which are the results of the additional iteration of adding a spatula of silica gel to the already purified 4:1 acid are also displayed. Bars and error bars represent mean ± standard deviation. For TPSiF, molecular sieve and silica gel 30:1 *n* = 3, for silica gel 4:1, *n* = 1 but analyzed in triplicate and for ultrapure water blanks, *n* = 10.

### Derivatization Optimization

The mixed effects model of the optimization factorial design showed significant interaction effects between acid-to-sample ratio and μmol of TPSiOH on derivatization efficiency (*p* = 0.03). Acid-to-sample ratio, TPSiOH, and derivatization time all had significant effects on derivatization efficiency on their own (*p* < 0.001), and the effects for all variables increased from the low to high level. Table 4 summarizes the result from the ANOVA of the mixed effects model, and an interaction plot of average derivatization efficiency over acid-to-sample ratio for the different levels of μmol TPSiOH is shown in Figure S1.^50^

**Table 4:** Results of Mixed-Effects Model ANOVA Testing Acid-to-Sample Ratio, μmol TPSiOH, Time, and Their Interaction Effects on the Derivatization Efficiency of F^–^

Factor	sum Sq	mean Sq	num DF	den DF	*F*- value	*P*-value
Acid-to-sample ratio	13,100	13,100	1	16	207	<0.001
TPSiOH	7700	7700	1	16	121	<0.001
Time	1960	1960	1	16	30.9	<0.001
Acid-to-sample ratio/TPSiOH	337	337	1	16	5.32	0.03
Acid-to-sample ratio/time	131	131	1	16	2.06	0.17
TPSiOH/time	2.43	2.43	1	16	0.0384	0.85

The center level (0.75 TPSiOH/1.5 acid-to-sample ratio) of the factorial design produced only derivatization efficiencies between the low and high levels and did not constitute any derivatization optima. In the initial design, even higher factor levels could have been used for the sake of finding the optimal combination of variable levels, which is why we chose to expand the acid-to-sample ratio to 3.3 and 4 for further testing. The results of all combinations of tested variables and levels are shown in Figure 2. A general trend in the derivatization efficiency was that increasing the acid-to-sample ratio reduced the necessary derivatization time to 20–40 min. Comparatively, other studies have reported necessary derivatization times of 1–3 h.^36,38,51^

**Figure 2:**
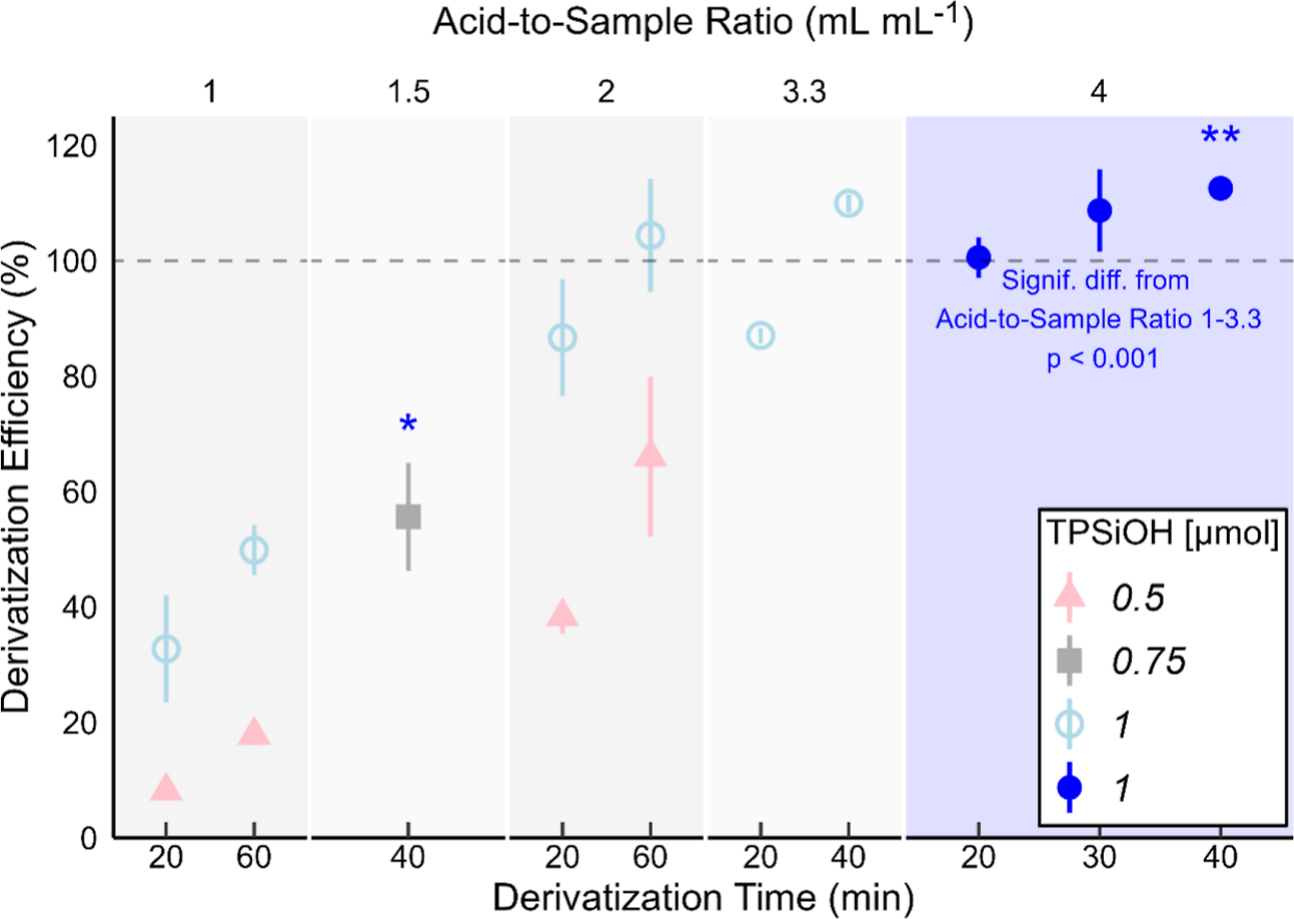
All combinations of variables tested with acid-to-sample ratios 1, 1.5, 2, 3.3, and 4; derivatization times 20, 30, 40, and 60 min; and amount of TPSiOH at 0.5, 0.75, and 1 μmol. Points and error bars represent mean ± standard deviation (*n* = 3, *n = 6, and **n = 2).

At acid-to-sample ratio 4, all derivatization times tested gave derivatization efficiencies close to 100% or above and were significantly different from tests carried out at lower ratios (Welch’s *t*-test, *p* < 0.001). Apparent derivatization efficiencies above 100% could be caused by matrix enhancing effects^52^ due to the different matrices of the derivatized sample solutions and the commercially sourced TPSiF standard solutions used for calibration. Hence, although derivatization efficiencies above 100% are not realistic, the results indicate that the efficiencies are very high and close to 100%.

Based on the results from the factorial experiment, the final optimized derivatization procedure was obtained and consisted of adding 1 mL of purified concentrated HNO_3_, 70 μL of 0.015 M TPSiOH, 250 μL of sample solution, and 300 μL of heptane to 1.7 mL Eppendorf tubes followed by 20–30 min vortexing. After vortexing, the tubes were centrifuged at 12 000 rpm for 10 min, and 150 μL of the heptane layer was transferred to GC analysis vials with 350 μL glass inserts and stored cold (+4 °C) until GC–MS/MS analysis.

### Method Validation

For the sake of matrix matching calibration standards with the samples, we investigated using a derivatized calibration curve based on the mixed anion IC solution instead of the commercially sourced TPSiF standard. Derivatized calibrations in the range from 5 to 1000 μg F^–^ L^–1^ yielded an *r* ^2^ of 0.998. If a narrower range (5–500 μg F^–^ L^–1^) was used, the *r* ^2^ was 0.9998 (Figure S1). The commercially sourced TPSiF calibration and derivatized calibration were strongly correlated (*r* ^2^ > 0.99, see Figure S3 and Table S1) and had a slope of 0.69–0.71 due to the different matrices. Results were comparable when accounting for the expected matrix deviations. The full derivatization includes both the derivatization step to form TPSiF and subsequent extraction into the heptane phase, steps that are unaccounted for when using the commercially sourced TPSiF for calibration. According to general practice^53,54^ and to properly account for these steps and matrix effects, we proceeded with using derivatized calibration curves. Using derivatized calibration curves, the LOD and LOQ were calculated to be 0.98 and 4.5 μg F^–^ L^–1^, respectively, based on the derivatization of 10 ultrapure water samples. In comparison, previous studies employing derivatization for F^–^ determination and GC or HPLC analysis reported detection limits of 7.6, 25, 6.8, and 3.2 μg F^–^ L^–1^ (GC-FID, GC–MS, HPLC-UV/vis, and headspace-GC–MS, respectively).^36,38,51^ It is possible that the LOD and LOQ could be lowered even further in this method by increasing the sample and acid volumes given that the derivatization and extraction to the heptane phase currently constitutes a slight dilution from 250 μL of sample to 300 μL of heptane.

Derivatization of the mixed anion IC standard showed good accuracy (95–99% of normalized standard concentration) and precision (relative standard deviation (RSD) from 0.7 to 5%) for 100–1000 μg F^–^ L^–1^, as shown in Figure 3. For the low, 5 μg F^–^ L^–1^ standard, precision was somewhat poorer (RSD = 7%), and the analyzed concentrations had a positive bias of 120 ± 8.4%. At 5 μg F^–^ L^–1^, this positive bias of 20% amounts to an increase in analyzed concentration of ∼1 μg F^–^ L^–1^, i.e., 6 ± 0.42 μg F^–^ L^–1^.

**Figure 3:**
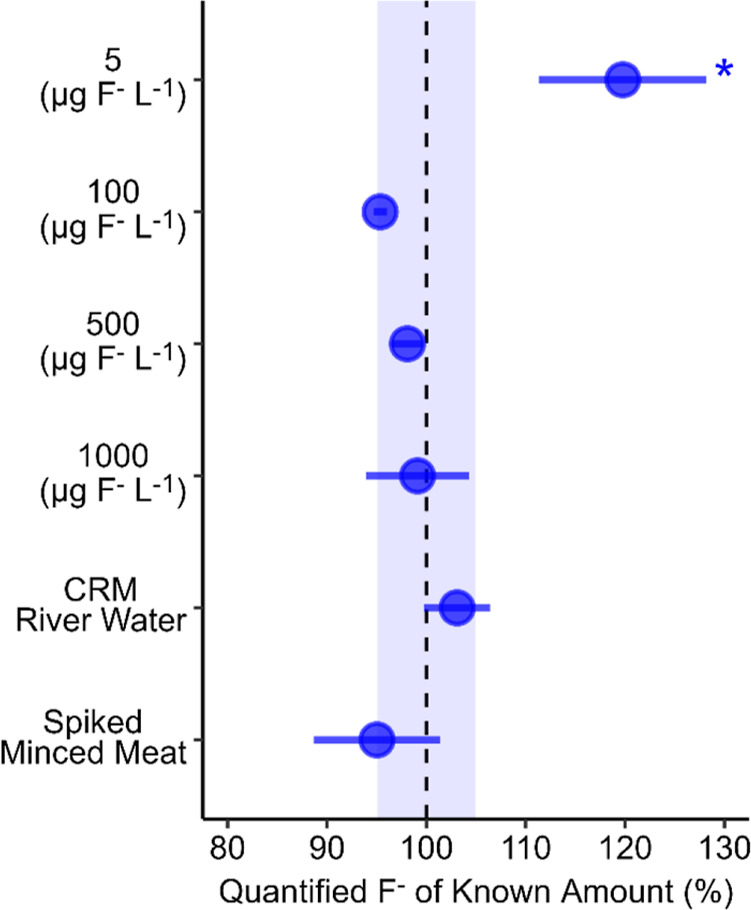
Precision and accuracy of mixed anion standards at 5, 100, 500, and 1000 μg F^–^L^–1^, the CRM River Water, and a spiked minced meat sample. Points and error bars represent mean ± standard deviation, *n* = 5 and *n = 4. The shaded area represents the 99% confidence interval based on the standard error of all measurements in the figure, normalized to 100%.

For the spiked minced meat samples (see the Supporting Information for details), 250 μL of aqueous extract was directly derivatized without any filtration or other cleanup procedure. The analyzed concentrations of the spiked minced meat were 95 ± 6% of spiked amounts of F^–^ (0.5 μg of F^–^ g^–1^ meat, accounting for background F^–^ meat concentrations of 0.072 ± 0.002 μg of F^–^ g^–1^ meat) (Figure 3). Derivatization and analysis of the CRM River Water gave 103 ± 3% of the certified reference value (1248 μg F^–^ L^–1^) (Figure 3). Furthermore, the CRM also contained chloride, nitrate, and sulfate at 31.3, 38, and 66.2 mg L^–1^, respectively. The obtained accuracy and precision of the fluoride analysis in the CRM demonstrate that the influence on derivatization efficiency is small from other anions even if present at high concentrations in relation to F^–^. The analysis of minced meat aqueous extract and the CRM River Water did not differ significantly from spiked and stated amounts of F^–^ (*p* = 0.31 and *p* = 0.10, respectively), indicating that the method produces accurate and reliable results even in the presence of complex biological and environmental matrices.

### F^–^ and EOF in Extracts

All sample extracts contained measurable levels of F^–^, implying that the term extractable fluorine^22^ more appropriately describes the fluorine content of the extracts, i.e., EF = EOF + F^–^. Actual EOF would then be calculated as the difference between EF and F^–^ as measured by the developed method. The F-mass balances of the extracts, using this approach (EOF = EF minus F^–^), are illustrated in Figure 4. Analysis of the river water tandem-SPE extracts (Oasis WAX extracts,^44^ ISOLUTE ENV+ and ISOLUTE 101), accounting for extraction blank contributions, showed that F^–^ was present at 29 ± 7.4, 30 ± 3.8, and 49 ± 29 μg F^–^ L^–1^ in the Oasis WAX, ISOLUTE ENV+, and ISOLUTE 101 extracts. In the Oasis WAX extracts, F^–^ accounted for 57 ± 15% of measured EF and for 24 ± 3 and 59 ± 35% of EF in the ISOLUTE ENV+ and ISOLUTE 101 extracts. The F^–^ in the Oasis WAX extracts are surprising since extensive rinsing steps were employed to reduce F^–^ retention during SPE. Additionally, EF varied between the tandem SPE extracts. The highest EF level (and calculated EOF) was observed in the ISOLUTE ENV+ extracts, indicating that some fluorinated compounds were not retained in the top Oasis WAX column but passed through and were retained on the ISOLUTE ENV+ and subsequent ISOLUTE 101 column. The higher EOF in the ISOLUTE ENV+ could be related to its affinity for polar organic compounds such as fluorinated pesticides^55,56^ and that the sampled river is the main drain for the Östergötland plain that is heavily dominated by agriculture. The AFFF soil extracts^27^ contained 61 ± 4.7 μg F^–^ L^–1^ which constituted 3 ± 0.2% of the measured EF content. The CRM Pike-perch extracts (Alkaline/Acidic MeOH^27^ and QuEChERSER–MeCN/H_2_O^47^) contained 17–90 μg F^–^ L^–1^, which accounted for 24–27% of EF. Similar approaches of correcting CIC measurements to yield a more accurate value of EOF have been done in studies analyzing fluorinated inorganic anions such as PF_6_^–^ and BF_4_^–^.^22^ However, incorporation of F^–^ determinations in the same extract volumes that is used for CIC-determinations has, to the best of our knowledge, not yet been reported.

**Figure 4:**
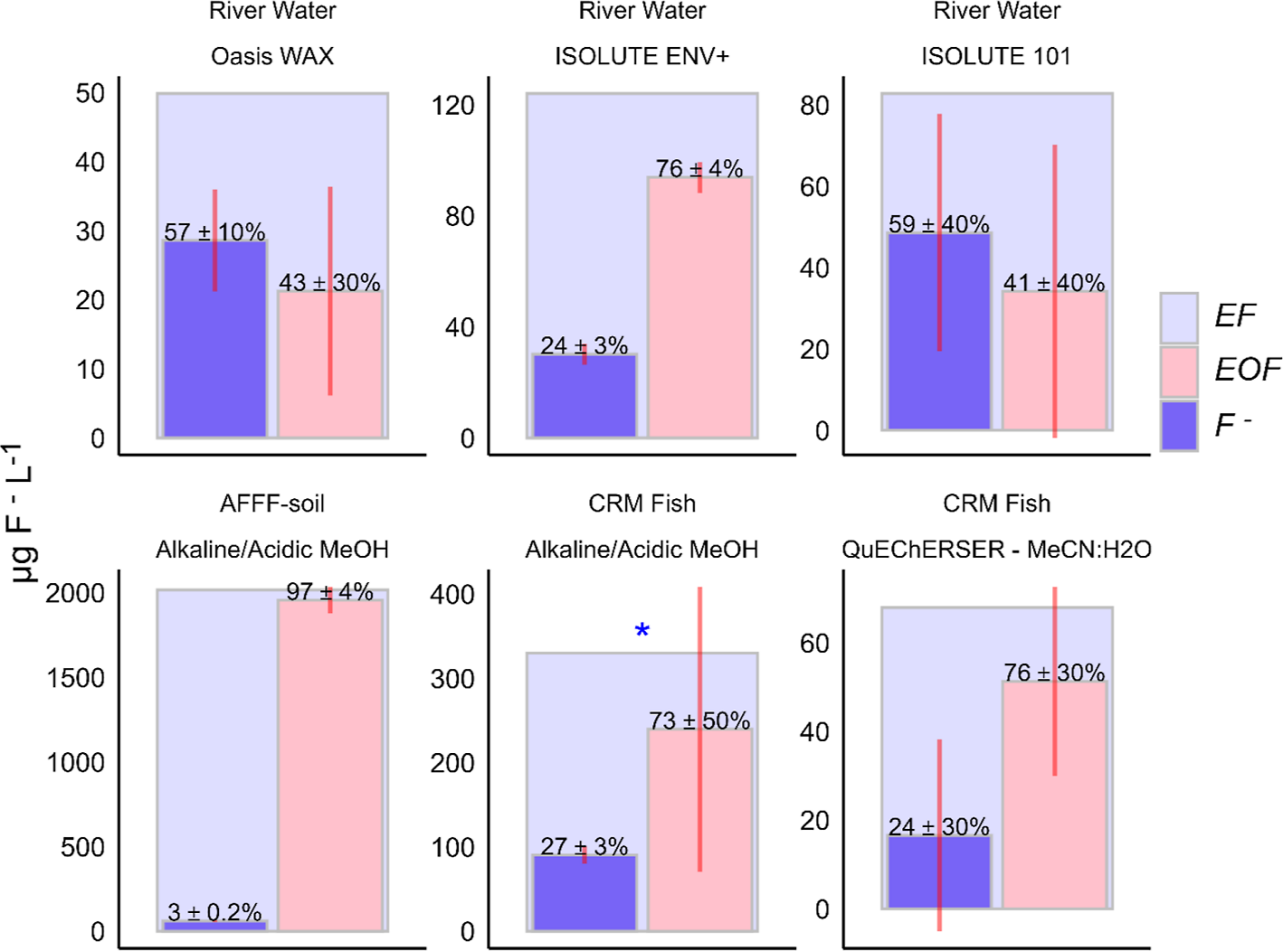
Fluorine mass balances of various extracts with average extractable fluorine (EF) (light purple broad bar) as measured by CIC and extractable OF (EOF, pink bar) calculated as the difference between EF and fluoride (F^–^, dark purple bar) in μg L^–1^. Displayed percentages represent the fraction of EF explained by measured F^–^ in each extract. Vertical bars represent one standard deviation of the mean, *n* = 3, *n = 2. Note that these bars reflect also the intrinsic variability among different samples. Empirical estimates of the total integrated uncertainty for EF, EOF, and F^–^ relative to the means are shown in Table S6.

The RSE for F^–^, EF, and EOF varied between extracts (see Table S6). For the F^–^ determinations, RSE was <10% in the ISOLUTE ENV+ and alkaline/acidic MeOH extracts (AFFF soil and CRM Pike perch). In contrast, the Oasis WAX extracts had a RSE of 15% for F^–^, and the QuEChERSER, MeCN/H_2_O extracts exhibited substantially higher variability with an RSE of 75%. For EOF, the mean RSE ranged from 3 to 61%. The higher RSEs for EOF were primarily due to the larger variability of the EF determinations as demonstrated by the consistently larger standard deviations (Figure 4) and standard errors of the mean of EF relative to F^–^ (Table S6). Thus, improving the precision of EF determinations would substantially improve the accuracy (i.e., lower the RSE) of EOF estimates.

### Incorporation into Workflows

The Oasis WAX protocol^44^ has been widely used for PFAS and EOF measurements to estimate unknown organofluorine compounds.^17,19,21,22,57^ The results of our developed method show that F^–^ can persist in extracts and constitute a significant portion of the measured EF. In cases where F mass balance calculations reveal large unexplained OF fractions, this method will be particularly useful to determine whether F^–^ constitutes parts of this unknown fraction.

EOF measurements have been proposed as a screening tool in workflows for potential PFAS contamination in different sample matrices.^58,59^ The previously suggested workflow has entailed the initial extraction of fluorinated compounds from samples and subsequent EOF analysis (with CIC or continuum source molecular absorption spectrometry^16,60^ (CS-MAS)). Samples shown to contain considerable amounts of EOF would then be subjected for further target analysis of PFAS to determine the contribution of known fluorinated compounds to the fluorine mass balance of the samples.^59^ Using such an approach, Aro et al. (2022)^58^ successfully differentiated between individuals exposed to AFFF contamination via drinking water and individuals of a control group with no known exposure to PFAS from drinking water through analysis of blood plasma extracts. Results showed that EOF analysis alone was able to make such a distinction. In such a workflow, the method developed in this study could complement the EF/EOF analysis by also determining if and to what extent F^–^ contributes to the determined EF/EOF. Ideally, the developed derivatization procedure and F^–^ measurement should be made in an aliquot of the same extract that is used for EF/EOF determination. Additional analysis on the same extract will require that adequate volumes of extract are available, especially if further analytical methods are to be employed downstream in the workflow, for example, with the total oxidizable precursor assay^61–65^ (TOP-assay) for the estimation of the presence of precursor compounds. Including duplicate extractions of samples,^58,59^ creating one replicate for target analysis (with isotopically labeled internal standards (IS) added during extraction) and one replicate (without added IS) for EF/EOF determination would provide enough extract for the small volumes used in the developed method and provide estimates of the potential F^–^ contribution to EF.

## Conclusions

A rapid derivatization method of F^–^ to TPSiF was successfully developed allowing for trace level determination of F^–^ in small aliquots (∼250 μL) of complex aqueous and solvent matrices using GC–MS/MS. In the developed method, there is no need to use perchloric acid that has previously been employed in studies utilizing TPSiOH (and other silanol-derivatives) for F^–^ determination,^12,36–38^ making it more user-friendly. In combination with decreased derivatization time, the method allows for a high throughput of samples, rendering it applicable for routine analysis. In this study, GC–MS/MS was used for analysis, but the method is also applicable for analysis with GC–MS and other detectors such as flame ionization and electron capture detectors^36^ (FID and ECD) increasing the methods applicability and versatility. A novel cleanup method for F^–^-contaminated concentrated HNO_3_ was also developed based on surface complexation of F^–^ to −Si–F using regular silica gel, effectively reducing background F^–^ levels to ∼1 μg F^–^ L^–1^ allowing for trace level determinations. The developed method was employed for the analysis of F^–^ in solvent extracts for PFAS and EOF analysis, clearly demonstrating that F^–^ contributes to the apparent EOF content in extracts when analyzed by CIC.

This study highlighted the risk that past analyses may have overestimated EOF concentrations, in some cases, as much as by ∼50%. Mitigating large biases are important for accurate monitoring of environmental concentrations and for the evaluation of remediation strategies to remove PFAS in soils and waters, particularly in light of current regulatory development concerning PFAS and EOF.^66^ Total sums of fluorine equivalents of targeted PFAS analysis, total organic fluorine, and EOF measurements have been discussed for establishing environmental quality standard (EQS) values in water, sediment, and biota within the EU Water Framework Directive.^66,67^ However, the risk of inclusion of inorganic fluorine species in these measurements has been highlighted as an important challenge limiting their use for establishing such threshold values.^66^ Incorporating the developed method for F^–^ determination in extracts would improve the estimation of the OF fraction by correcting for F^–^ content. As such, we recommend that F^–^ determinations of extracts are routinely employed to improve the accuracy of the fluorine mass balance calculations and avoid overestimation of the EOF content.

## Supplementary Material



## Notes

F^–^ removal from concentrated HNO_3_, additional chemical used, linearity during method validation, and extraction procedures (PDF)

## Abbreviations

AFFF: aqueous film-forming foam
EF: extractable fluorine
F^–^: fluoride
F: fluorine
EOF: extractable organic fluorine
GC–MS/MS: gas-chromatography tandem mass spectrometry
HNO_3_: nitric acid
MeCN: acetonitrile
MeOH: methanol
PFAS: perfluoro alkyl substances
SPE: solid-phase extraction
TPSiF: triphenylfluorosilane
TPSiOH: triphenylsilanol
