# Supplementary material for: Toward Accurate Estimation of Extractable Organic Fluorine by Analysis of Fluoride Contamination in Extracts Using Gas Chromatography
Source: Anal Chem. 2026 Apr 2;98(14):10511–20. doi: 10.1021/acs.analchem.5c07147 (PMC13084625; doi:10.1021/acs.analchem.5c07147)
Supplement: Supplementary file 1 [file ac5c07147_si_001.pdf]

Supporting Information for

Towards accurate estimation of extractable organic fluorine (EOF) by analysis of fluoride contamination in extracts using gas chromatography

Johannes Kikuchi-McIntosh[a]*, *Malin Montelius*[b], *Gustav Sporre*[a], *David Bastviken*[a] *and Teresia Svensson*[a]

[a] Department of Thematic Studies – Environmental Change, Linköping University, Linköping, SE-581 83, Sweden

[b] Swedish Geotechnical Institute (SGI), Linköping, SE-581 93, Sweden

*Email: johannes.kikuchi@liu.se

Table of contents for Supporting Information 1

Additional Chemicals and Materials 3

Theoretical basis of F^- removal through surface complexation 4

F^- removal from concentrated HNO_3 4

F- spiked minced meat and aqueous extraction 5

Extraction Procedures 5

Water - SPE-extraction 5

Soil - Methanol (acidic/alkaline) extraction 7

Fish - acetonitrile (QuEChERSER) extraction 8

Fish - alkaline and acidic extraction 8

Supplementary Figures 9

Figure S1 Interaction Plot form Factorial Design 9

Figure S2 Derivatized Calibration Curve 10

Figure S3 Regression analysis: Derivatized standards over sourced TPSiF standards 11

Table S1 Regression analysis summary output. 11

Data Tables 12

Table S2.Data from Fluoride Removal Tests in concentrated HNO_3 12

Table S3 Data from Factorial Design Experiment 13

Table S4 Data from Precsision and Accuracy Experiment 14

Table S5 Data from Massbalance Analysis and Calculations of Extract EOF 15

Table S6. Uncertainty as SEM and RSE of F fractions in Extracts. 17

Additional Chemicals and Materials

Methanol (MeOH, Supelco Lichrosolv) and acetonitrile (MeCN, Supelco Lichrosolv) were LC-MS grade and purchased from VWR. Other chemicals and material used in the various extraction procedures were ammonium hydroxide for HPLC (25 % NH_4OH, LiChropur, Supelco), concentrated hydrochloric acid, 32 % (HCl, Fluka, Honeywell), acetic acid (>99.8 purity, Sigma-Aldrich), solid phase extraction (SPE)-cartridges Oasis WAX (150 mg, 30 µm, 6 cc, Waters), ISOLUTE ENV+ (200 mg, 6 mL, Biotage) and ISOLUTE 101 (200 mg, 6 mL, Biotage). ENVI-CARB SPE Bulk (Supelclean) was used for extract clean-up when needed and was purchased from Sigma-Aldrich. Solid-liquid extractions were performed in 15 and 50 mL PP-tubes (Sarstedt) and smaller extract volumes were centrifuged in 1.7 mL Eppendorf-tubes. All plasticware was rinsed three times with MeOH before use. Glassware and glass Pasteur pipettes were combusted at 450 ^{o}C (>2h) and then rinsed three times with MeOH before use.

Theoretical basis of F^- removal through surface complexation

The idea of using silica for F^- removal came from Stumm (1992) [1] and is based on ligand exchange of anions on oxide surfaces, an example of which are silicates. Silicates in aqueous solutions at low pH tend to have fully protonated surface hydroxyl-groups (or silanol-groups, Si-OH or $Si\text{-}OH_2^+$) that can be exchanged with anions and weak acids in solution. In these surface complexation reactions, the central surface Si-atom acts as a Lewis acid that receives a pair of electrons from the anion or weak acid. Accordingly, silica gel (SiO_2) should be able to undergo such surface reactions in aqueous solutions at low pH while F^-, being both an anion and a weak acid, should be able to act as a Lewis base that donates an electron pair to the electron deficient -Si-OH-complex. This would facilitate ligand exchange with the surface hydroxyl groups and generate surface Si-F-complexes and OH^- in solution and effectively remove F^- from solution.

F^- removal from concentrated HNO_3

Direct derivatization of F^- to TPSiF and subsequent extraction of the acid was tested by adding 1 mL 0.015 M TPSiOH to 9 ml of concentrated HNO_3 and shaking on an orbital shaker for 1 hour. The acid was then extracted twice with 1 ml heptane which was discarded. A 3 ml aliquot of the acid was then transferred to a 15 mL PP-tube followed by 1 hour derivatization with 200 μL 0.015 M TPSiOH and 1 mL of heptane. After derivatization the tube was centrifuged and the heptane layer transferred to a GC-analysis vial. A second heptane extraction was then performed on the acid and the two heptane aliquots were combined and analyzed. Molecular Sieve 3 Å (MS, Thermo Scientific) and Silica Gel for column chromatography (SG, Supelco, 60 μm) were tested for the surface-complexation approach by mixing with concentrated HNO_3 at a ratio of

30:1 (9 mL conc HNO_3:0.3 g SiO_2) in 15 mL PP-tubes. Tubes were then sonicated for 2 min, centrifuged at 10 000 rpm for 5 min after which 3 mL of the acid was transferred to a new 15 mL tube. Derivatization of the 3 mL aliquots were then performed and analyzed as previously described. The 4:1 treatment with silica gel and the additional test with 40 mL 4:1 treated acid and a spatula of silica gel were handled like the 30:1 treatments and derivatized as previously described.

F- spiked minced meat and aqueous extraction

To 5 ± 0.1 g of minced meat was added 500 µL 5 mg L^{-1} of mixed anion IC-standard. Samples were let to equilibrate overnight and were extracted three times with 5 mL ultrapure water to a total volume of 15 mL with ultrasonication followed by centrifugation at 12 000 rpm for 10 min. Unspiked minced meat was also extracted and analyzed to get the background concentration of F^- of the meat. Percent recovery was then assessed by subtracting the background concentrations of F^- from the analyzed concentrations of the spiked meat and comparing with the spiked amounts of F^-.

Extraction Procedures

Water - SPE-extraction

For the river water, SPE was performed with Oasis WAX columns according to the PFAS/EOF-method by Miyake et al. (2007)[2]. This method includes an extensive rinsing step to maximize removal of F^- prior to PFAS-elution and subsequent EOF-analysis with CIC. To investigate F^- content in extracts from other types of SPE-columns we stacked an ISOLUTE ENV+ and an ISOLUTE 101 column underneath the Oasis Wax column, performing SPE-

extraction in tandem. In short, triplicate samples of 5 L river water were acidified to pH 2 with silica-gel-cleaned HNO_3 (~ 3 mL) followed by filtration over pre-combusted glass fiber filters (~450 ^oC, > 2h). Each sample was then loaded onto a stack of preconditioned SPE-columns (from the top; Oasis WAX, ISOLUTE ENV+ and ISOLUTE 101) at a flow rate of approximately 3.5 mL min^{-1}. Each type of SPE-column underwent separate processes for preconditioning, rinsing, drying and elution. The Oasis WAX columns were subjected to the following procedure according to Miyake et al. 2007[2]. First, 4 mL 0.1 % NH_4OH in MeOH was loaded and eluted followed by 4 mL of MeOH and 4 mL of ultrapure-water acidified to pH 2. The rinsing step after sample loading to remove F^- consisted of first adding 20 mL 0.01 % NH_4OH in ultrapure water followed by four 10 mL additions of ultrapure water. A gentle stream of N_2-gas was then used to dry the Oasis Wax columns before elution with 10 mL 0.1 % NH_4OH in MeOH into 15 mL PP-tubes. The ISOLUTE ENV+ and ISOLUTE 101-columns were subjected to the following processing steps according to application notes from the manufacturer[3,4]; preconditioning with 4 mL 0.1 % formic acid in MeOH followed by 4 mL of ultrapure water adjusted to pH 2, rinsing with 10 mL 0.1 % formic acid in ultrapure water, drying by gravity, elution with 10 mL of 0.1 % formic acid in MeOH into 15 mL PP-tubes. In the elution step an aliquot of the elution volume was first allowed to soak into the sorbent bed before adding the remaining elution volume. All extracts were then evaporated to < 0.5 mL under N_2-flow at 30 ^oC (Zip-Vap) and quantitatively transferred to 1.7 mL Eppendorf-tubes. All extracts were then adjusted to 1.5 mL total volume with pure MeOH and stored in freezer (-20 ^oC) until analysis.

Soil - Methanol (acidic/alkaline) extraction

The AFFF-impacted soil had previously been sieved < 2 mm and homogenized extensively. Aliquots of the soil corresponding to 2 g dry weight were freeze dried in 50 mL PP-tubes prior to extraction with a modified version of the enhanced alkaline and acidic PFAS extraction method by Nickerson et al. (2020)[5]. In short, 4 mL of 0.1 M NH_4OH in MeOH was added to each test tube, vortexed for 30 s and then sonicated for 15 min at 30°C followed by centrifugation at 12 000 rpm x 15 min. The supernatant was decanted into a new 50 mL PP-tube and the procedure was repeated once more collecting the alkaline extracts in the same tube. Following the two alkaline extractions, 4 mL of 0.5 M HCl in MeOH was added to the remaining soil pellet, vortexed for 30 s, sonicated and centrifuged as previously described and decanted into a new 50 mL PP-tube. The acidic extraction was repeated once more and the acidic extracts pooled. The alkaline and acidic extracts were then combined in yet another 50 mL PP-tube with pre-weighed 20 mg of ENVI-CARB powder, vortexed for 30 s and put on an orbital shaker for 30 min at 180 rpm. Extracts were then neutralized by addition of 0.5 mL 25 % NH_4OH-solution. As mentioned by Nickerson et al. (2020), a lot of precipitate formed when combining the extracts and during the neutralization step, so extracts were then centrifuged at 12 000 rpm at 15 min and transferred to new 50 mL PP-tubes. Extract volumes were then evaporated to near dryness and reconstituted in 1.5 mL 1 % acetic acid in MeOH and then transferred to Eppendorf-tubes and put in the freezer (-20 °C). After ~2 h the tubes were centrifuged for 10 min at 17 000 g and the supernatant transferred to new Eppendorf-tubes. A lot more precipitate formed during

freezing but the resulting supernatant after centrifuging was very clear and colorless. Soil extracts were stored in freezer until analysis.

Fish - acetonitrile (QuEChERSER) extraction

The fish sample was a perch reference sample for PFAS-analysis (CRM IRMM 427 Pike-perch). This sample was extracted according to a modified version of the QuEChERSER-method (more than QuEChERS) for PFAS-analysis in foods by Taylor and Sapozhnikova (2022)[6]. The original method comprises of a splitting of extracts into an LC- and a GC-portion with subsequent clean-up procedures for the GC-extract. In this study, however, we were interested in the possibility of analyzing F^- in various types of complex sample extract matrices and as such the clean-up procedures described for the GC-extract were omitted. In short, aliquots of ~1 g of perch sample were weighed into 15 mL PP-tubes and 5 mL of 4:1 MeCN:Water was added followed by 10 min vortexing. Then, 1 g of salt (4:1, $MgSO_4$:NaCl (wt/wt)) was added to each tube, shaken by hand for 1 min and then centrifuged at 3711 g for 3 min. The MeCN-layer was then transferred to a new 15 mL PP-tube and stored in freezer until analysis.

Fish - alkaline and acidic extraction

In addition, the alkaline and acidic extraction procedure by Nickerson et al. (2020) was tested on the perch reference sample. The same modified procedure as previously described for soil was used for this test but with ~1 g of perch sample. Extracts were stored in freezer until analysis.

Supplementary Figures

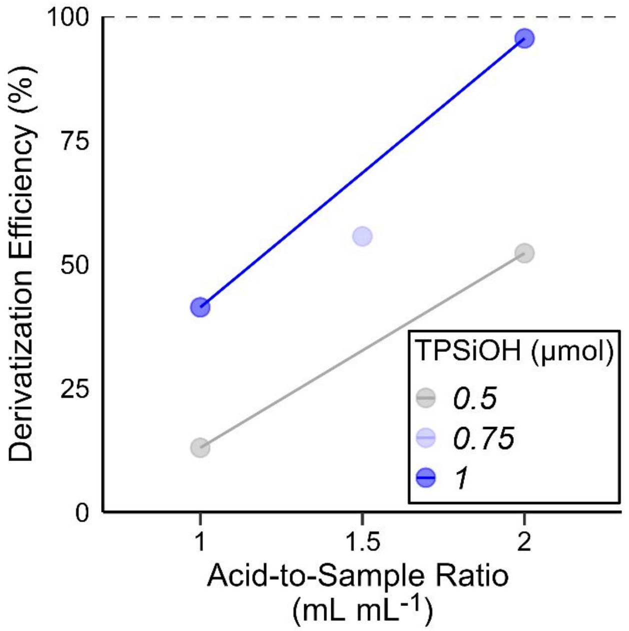

Figure S1. Interaction plot of average derivatization efficiency (%) over increasing Acid-to-Sample Ratio (1, 1.5 and 2 mL mL^{-1}) for the low-, center- and high levels of TPSiOH (0.5, 0.75 and 1 μmol, respectively).

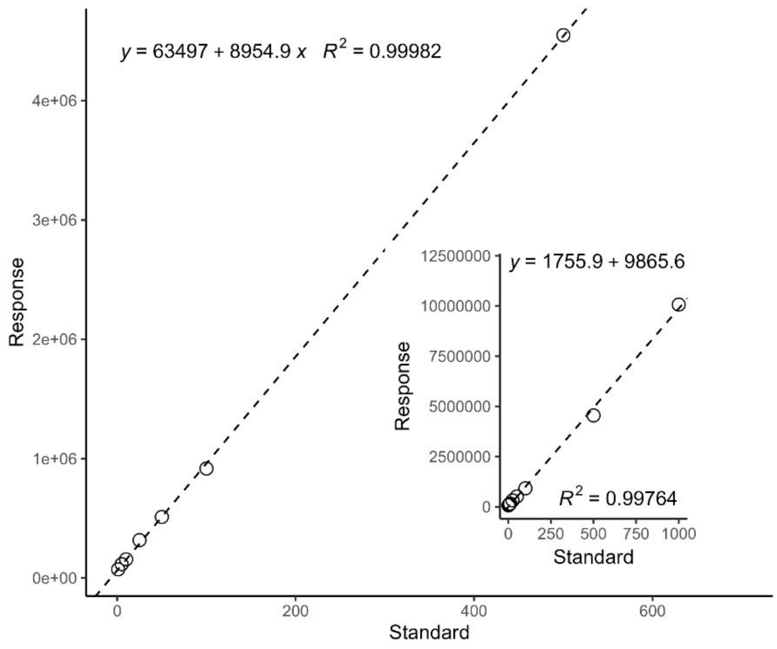

Figure S2. Derivatized calibration curves with standards 5, 10, 25, 50, 100, 500 µg F^- L^{-1} and 5, 10, 25, 50, 100, 500 and 1000 µg F^- L^{-1} and associated correlation coefficients (R^2).

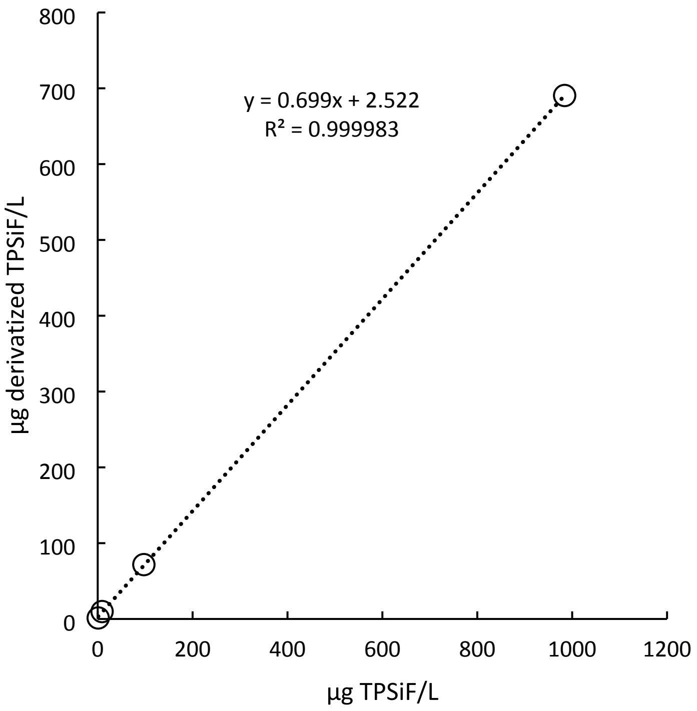

Figure S3. Regression of derivatized calibration standards (1, 10, 100, 500, 1000 µg F^{-} L^{-1}, quantified by commercially sourced TPSiF calibration) over the commercially sourced TPSiF standards (1, 10, 100, 500, 1000 µg TPSiF L^{-1}).

Table S1. Summary output of regression analysis of quantified concentrations of derivatized calibration standards over commercially sourced TPSiF standards.

	Coefficients	*Standard Error*	*t Stat*	*P-value*	*Lower 95%*	*Upper 95%*
Intercept	2.52	1.01	2.51	0.13	-1.81	6.85
Slope	0.70	0.00	343.68	0.00001	0.69	0.71

Data Tables

Table S2. Tested F^- removal procedures from concentrated HNO_3 and their analyzed concentrations. The current blank samples were made with 40 mL of the SG4:1 acid with an added spatula of silica gel and their concentration represents the current method blank levels. N.D = not detected.

Removal Procedure / Sample ID	TPSiOH (0.15 M) / Silica	HNO_3	Concentration
	µL / g	mL	µg F^- L^{-1}
TPSiOH	1000	9	560
TPSiOH	1000	9	1500
TPSiOH	1000	9	1400
MS30:1	0.30	9	180
MS30:1	0.30	9	260
MS30:1	0.29	9	180
SG30:1	0.30	9	34
SG30:1	0.30	9	43
SG30:1	0.30	9	81
SG4:1	121	400	9
SG4:1	121	400	25
SG4:1	121	400	11
Current Blank 1			0.9
Current Blank 2			N.D
Current Blank 3			0.3
Current Blank 4			1.1
Current Blank 5			1.2
Current Blank 6			0.2
Current Blank 7			0.8
Current Blank 8			0.4
Current Blank 9			1.5
Current Blank 10			0.9

Table S3. Layout of the factorial design for optimization of the derivatization procedure with analyzed concentrations and calculated derivatization efficiency.

Block	ID	Acid-to-Sample Ratio	TPSiOH	Time	Concentration	Theoretical Concentration	Derivatization Efficiency
		mL mL^{-1}	µmol	min	µg F^{-} L^{-1}	µg F^{-} L^{-1}	%
I	Run1_S19	1	1	60	48	100	48
I	Run2_S21	1	1	60	47	100	47
I	Run3_S11	2	1	20	90	100	90
I	Run4_S17	2	0.5	60	58	100	58
I	Run5_S26	1.5	0.75	40	62	100	62
I	Run6_S10	2	1	20	75	100	75
I	Run7_S20_2	1	1	60	55	100	55
I	Run8_S12	2	1	20	94	100	94
I	Run9_S18_3	2	0.5	60	82	100	82
I	Run10_S1	1	0.5	20	7	100	7
I	Run11_S27	1.5	0.75	40	71	100	71
I	Run12_S2	1	0.5	20	8	100	8
I	Run13_S3	1	0.5	20	9	100	9
I	Run14_S25	1.5	0.75	40	55	100	55
I	Run15_S16	2	0.5	60	59	100	59
II	Run16_S14	1	0.5	60	18	100	18
II	Run17_S30	1.5	0.75	40	48	100	48
II	Run18_S24	2	1	60	107	100	107
II	Run19_S23	2	1	60	113	100	113
II	Run20_S15	1	0.5	60	19	100	19
II	Run21_S6	2	0.5	20	38	100	38
II	Run22_S4	2	0.5	20	42	100	42
II	Run23_S29	1.5	0.75	40	49	100	49
II	Run24_S7	1	1	20	23	100	23
II	Run25-S5	2	0.5	20	36	100	36
II	Run26_S22	2	1	60	94	100	94
II	Run27_S2	1	1	20	34	100	34
II	Run28_S28	1.5	0.75	40	48	100	48

II	Run29_S13	1	0.5	60	16	100	16
II	Run30_S9	1	1	20	41	100	41

Table S4. Analyzed concentrations of the precision and accuracy tests with derivatization of the mixed anion standard SS-9195S (Spectrascan, Teknolab), certified reference material LGC6025 River Water – Anions (LGCStandards) and F^- spiked minced meat at a concentration of 0.5 µg F^- g^{-1}.

Sample ID	Concentration	Theoretical Concentration	Accuracy
	µg F^- L^{-1} / µg F^- g^{-1}	µg F^- L^{-1} / µg F^- g^{-1}	% of theoretical
5	6.2	5	124
5	5.7	5	114
5	4.6	5	93
5	6.5	5	130
5	5.6	5	112
100	95	100	95
100	96	100	96
100	96	100	96
100	94	100	94
100	95	100	95
500	504	500	101
500	490	500	98
500	491	500	98
500	486	500	97
500	483	500	97
1000	1062	1000	106
1000	983	1000	98
1000	954	1000	95
1000	1023	1000	102
1000	934	1000	93
CRM River Water	1235	1248	99
CRM River Water	1271	1248	102
CRM River Water	1272	1248	102
CRM River Water	1341	1248	107

CRM River Water	1314	1248	105
Spiked Minced Meat	0.46	0.5	91
Spiked Minced Meat	0.51	0.5	102
Spiked Minced Meat	0.46	0.5	92

Table S5. Analyzed concentrations of F^-, EF and calculated EOF in the various extracts (μg L^{-1}).

Sample ID	Sample/Blank	Sample Type	Extraction	Fluoride	Extractable Fluorine	Extractable Organic Fluorine
				μg L^{-1}	μg L^{-1}	μg L^{-1}
AFFF1A	Sample	AFFF-soil	Alkaline/Acidic MeOH	67	2160	2090
AFFF1B	Sample	AFFF-soil	Alkaline/Acidic MeOH	74	2270	2190
AFFF1C	Sample	AFFF-soil	Alkaline/Acidic MeOH	76	2320	2240
AFFFBLA	Blank	AFFF-soil	Alkaline/Acidic MeOH	11	240	220
AFFFBLB	Blank	AFFF-soil	Alkaline/Acidic MeOH	12	280	270
AFFFBLC	Blank	AFFF-soil	Alkaline/Acidic MeOH	12	180	170
Svartån WAX A	Sample	River Water	SPE Oasis Wax	96	140	40
Svartån WAX B	Sample	River Water	SPE Oasis Wax	81	100	20
Svartån WAX C	Sample	River Water	SPE Oasis Wax	89	100	10
Svartån ENV+ A	Sample	River Water	ISOLUTE ENV+	51	200	150
Svartån ENV+ B	Sample	River Water	ISOLUTE ENV+	48	210	160
Svartån ENV+ C	Sample	River Water	ISOLUTE ENV+	56	210	150
Svartån 101 A	Sample	River Water	ISOLUTE 101	110	150	50
Svartån 101 B	Sample	River Water	ISOLUTE 101	72	190	110
Svartån 101 C	Sample	River Water	ISOLUTE 101	52	110	60
Blank WAX A	Blank	River Water	SPE Oasis Wax	50	50	0
Blank WAX B	Blank	River Water	SPE Oasis Wax	63	80	20
Blank WAX C	Blank	River Water	SPE Oasis Wax	68	60	-
Blank ENV+ A	Blank	River Water	ISOLUTE ENV+	18	100	80
Blank ENV+ B	Blank	River Water	ISOLUTE ENV+	23	70	40
Blank ENV+ C	Blank	River Water	ISOLUTE ENV+	24	80	50
Blank 101 A	Blank	River Water	ISOLUTE 101	21	50	30
Blank 101 B	Blank	River Water	ISOLUTE 101	33	60	30
Blank 101 C	Blank	River Water	ISOLUTE 101	34	100	60
CRM Perch-pike B	Sample	CRM Fish	Alkaline/Acidic MeOH	96	1630	1540
CRM Perch-pike C	Sample	CRM Fish	Alkaline/Acidic MeOH	110	1410	1300
Blank A	Blank	CRM Fish	Alkaline/Acidic MeOH	12	650	640
Blank C	Blank	CRM Fish	Alkaline/Acidic MeOH	12	1100	1090
CRM Perch-pike A	Sample	CRM Fish	QuEChERSER - MeCN:H2O	13	400	390
CRM Perch-pike B	Sample	CRM Fish	QuEChERSER - MeCN:H2O	13	360	350

CRM Perch-pike C	Sample	CRM Fish	QuEChERSER - MeCN:H2O	50	420	370
Blank B	Blank	CRM Fish	QuEChERSER - MeCN:H2O	10	320	310
Blank C	Blank	CRM Fish	QuEChERSER - MeCN:H2O	7	340	330

Table S6. Standard error of the mean (µg F L^{-1}) and relative standard error of the mean (%) for F^-, EF and EOF for each extract type.

Extraction	Type	Standard Error of the Mean			Relative Standard Error of the Mean		
		F^-	EF	EOF	F^-	EF	EOF
		µg F L^{-1}	µg F L^{-1}	µg F L^{-1}	%	%	%
SPE Oasis Wax	River Water	4	12	9	15	24	41
ISOLUTE ENV+	River Water	2	3	3	7	2	3
ISOLUTE 101	River Water	17	21	21	35	25	61
Alkaline/Acidic MeOH	AFFF-soil	3	48	45	4	2	2
Alkaline/Acidic MeOH	CRM Fish	7	112	119	8	34	50
QuEChERSER - MeCN:H2O	CRM Fish	12	18	12	75	26	24

REFERENCES

(1) Stumm, W. *Chemistry of the Solid-Water Interface: Processes at the Mineral-Water and Particle-Water Interface in Natural Systems*; New York, NY (United States); John Wiley Sons: United States, 1992.

(2) Miyake, Y.; Yamashita, N.; Rostkowski, P.; So, M. K.; Taniyasu, S.; Lam, P. K. S.; Kannan, K. Determination of Trace Levels of Total Fluorine in Water Using Combustion Ion Chromatography for Fluorine: A Mass Balance Approach to Determine Individual Perfluorinated Chemicals in Water. *Journal of Chromatography A* **2007**, *1143* (1–2), 98–104. https://doi.org/10.1016/j.chroma.2006.12.071.

(3) Biotage. TN119.V.1 ISOLUTE 101 Aqueous, 2020.

(4) Biotage. TN109.V.1 Meth Dev Guidelines ENV+, 2020.

(5) Nickerson, A.; Maizel, A. C.; Kulkarni, P. R.; Adamson, D. T.; Kornuc, J. J.; Higgins, C. P. Enhanced Extraction of AFFF-Associated PFASs from Source Zone Soils. *Environ. Sci. Technol.* **2020**, *54* (8), 4952–4962. https://doi.org/10.1021/acs.est.0c00792.

(6) Taylor, R. B.; Sapozhnikova, Y. Comparison and Validation of the QuEChERSER Mega-Method for Determination of per- and Polyfluoroalkyl Substances in Foods by Liquid Chromatography with High-Resolution and Triple Quadrupole Mass Spectrometry. *Analytica Chimica Acta* **2022**, *1230*, 340400. https://doi.org/10.1016/j.aca.2022.340400.
